# Dietary Hydrolyzed Yeast Improves Growth, Gut Health, and Selective Gene Expression of Nile Tilapia (*Oreochromis niloticus*)

**DOI:** 10.1155/anu/7934851

**Published:** 2025-09-05

**Authors:** Mohiuddin Amirul Kabir Chowdhury, Ankon Lahiry, Md. Lifat Rahi, Md. Amzad Hossain, Gustavo de Aguiar, Graziela Alves, Aung Tun Aye, Rajib Dutta, Melina Aparecida Bonato, Albert G. J. Tacon

**Affiliations:** ^1^Maverick Innovation, Kaliakair, Gazipur, Bangladesh; ^2^Fisheries and Marine Resource Technology, Khulna University, Khulna, Bangladesh; ^3^Department of Aquaculture, Gazipur Agricultural University, Gazipur, Bangladesh; ^4^ICC Animal Nutrition, Sao Paulo, Brazil; ^5^Agro Solutions, Gulshan, Dhaka, Bangladesh; ^6^Aquahanna LLC, 93 Pinana St., Kailua 96734, Hawaii, USA

**Keywords:** gene expression, growth, hydrolyzed yeast, Nile tilapia

## Abstract

The effects of graded levels of hydrolyzed yeast (HY) supplementation (0.0, 0.5, 1.0, and 2.0 g/kg, i.e., Control, HY0.5, HY1.0, HY2.0, respectively) on growth performance, gut health, and immune responses of juvenile Nile tilapia (*Oreochromis niloticus*) were assessed in this study. The experiment was conducted in a completely randomized design for 14 weeks, where the treatments were distributed in 16 300-L tanks with four replicates each. Despite no significant differences, the final body weight and weight gain were numerically higher in treatments containing HY (44.7 and 34.7 g, 43.5 and 33.5 g, and 45.5 and 35.5 g in HY0.5, HY1.0, and HY2.0, respectively). Feed efficiency (FE) was improved linearly (*p* < 0.05) with increasing dietary HY level (0.65, 0.70, and 0.72, respectively). Similarly, there was also a significant linear relationship between protein deposition (PD), as well as protein and energy retention efficiency (ERE), with the increasing dietary HY level. Among the blood parameters, only the hematocrit (HCT) value was significantly lower in HY1.0 and HY2.0 compared to the control and HY0.5 treatments. Gut histology showed significantly higher villi length in fish fed HY2.0 diets (795 ± 89.6 µm) compared to those fed the control diet (504 ± 80.7 µm). The average surface volume (SV) of the villi was also higher in tilapia fed HY0.5, HY1.0, and HY2.0 diets (0.025, 0.026, and 0.038 mm^3^, respectively) compared to the control diet (0.021 mm^3^). All four target genes were significantly upregulated in HY1.0 and HY2.0 treatments. The expression of the genes supporting growth and ATP production, insulin-like growth factor-1 (IGF-1) and glyceraldehyde-3-phosphate (G-3-P), respectively, was significantly improved, as well as the expression of the immune-related gene, hepcidin. The expression of ghrelin also showed a significant increase with increasing HY levels. It can be concluded that the HY supplementation improved feed utilization, gut health, nutrient absorption capacity, and immunity in Nile tilapia.

## 1. Introduction

Nile tilapia (*Oreochromis niloticus*) is one of the most popular farmed fish species worldwide, ranking third with an 8.3% share in global aquaculture production [1, 2]. Despite impressive growth and wide acceptance, tilapia aquaculture has been facing many challenges such as the costs of feed and frequent disease outbreaks [3]. These challenges are particularly evident in intensively stocked systems with inadequate biosecurity and water quality management, where they are naturally susceptible to bacterial, fungal, and parasitic diseases [4, 5]. A recent report suggested that tilapia farmers in Bangladesh face an average of 23.2% unusual mortality, which resulted in frequent use of antibiotics by those farmers [5]. The high mortality prompted excessive use of antibiotics in most Asian countries, particulary in Vietnam, China, Bangladesh [6], and the Philippines [7].

The growing global awareness of antimicrobial resistance increased the search for natural alternatives to antibiotics [8]. A variety of natural feed additives, including enzymes [9–11], probiotic and prebiotic [12–14], organic acids and their salts [15, 16], and phytogenic compounds or essential oils [17] have been shown to positively affect the growth performance, immunity, and gut health of tilapia, ultimately lowering the need for antibiotics.

Yeast-derived feed additives, including nucleotides, beta-glucans, mannan oligosaccharides (MOSs), and chitins, have been recognized for their efficacy in improving intestinal health, controlling pathogens, and enhancing innate immune response [18, 19]. These components, either working independently or synergistically, may promote growth depending on the inclusion level and formulation [20]. Nucleotides, part of the intracellular fluid, have been investigated in various fish species, including grouper (*Epinephelus malabaricus*), Caspian brown trout (*Salmo trutta caspius*), and rainbow trout (*Oncorhynchus mykiss*) [21–23]. Although not classified as essential nutrients, dietary nucleotide supplementation has shown to improve physiological functions, morphological profiles, and intestinal microbiota composition [18, 24, 25], while also enhancing immune responses during stress and supporting reproductive system development in fish [26, 27]. Beta-glucans (primarily β-1,3 and 1,6 glucans), derived from the yeast cell wall, serve as biological response modifiers that activate phagocytes to produce cytokines, thereby stimulating the innate immune response [28], which is essential during early growth phases, reproductive phases, and under environmental and physiological stress [29–31]. Similarly, MOS, derived from yeast cell wall, is one of the polysaccharides widely used in aquafeed (typically included at 0.4%–0.6%) to enhance the immunological and gastrointestinal functions [32–34]. These polysaccharides can prevent pathogen colonization in the gut by binding with the Type 1 fimbriae of some pathogens [35, 36] and then excreted with the fecal material [37]. Although less known, chitin is considered a prebiotic and possess immunostimulatory properties when used in moderation [19].

Hilyses (referred to as hydrolyzed yeast [HY]) is a yeast-derived additive produced from the enzymatic hydrolysis of *Saccharomyces cerevisiae*, utilized in the fermentation of sugarcane for ethanol production. During the process, the yeast undergoes cell autolysis and intracellular content hydrolysis. This final product is highly digestible, containing free nucleotides and nucleosides, amino acids, peptides and polypeptides, glutamine, chitin, MOS, and high levels of β-glucans [20, 38]. This valorization of agro-industrial waste aligns with circular economy principles, promoting sustainability in aquafeed formulation by transforming low-value byproducts into functional ingredients [39–41]. Therefore, the objective of the present study was to assess the effects of graded-level of the HY on growth, gut health, and selective gene expression of Nile tilapia (*Oreochromis niloticus*), with the aim of establishing an optimal inclusion level through dose–response assessment.

## 2. Materials and Methods

### 2.1. Experimental Site and Ethical Statement

This trial was carried out at the recirculating aquaculture system (RAS) unit under the Aqua Innovation Research Division of Maverick Innovation, Gazipur, Bangladesh. All the experimental protocols were approved by the Animal Care Committee of Maverick Innovation (ACC/MI/2025/01).

### 2.2. Water Quality Parameters

Throughout the experimental period, all water quality parameters, except temperature, were maintained within the optimum ranges for Nile tilapia. Specifically, the average dissolved oxygen (DO), pH, and ammonia levels were maintained at 5.8 ± 0.1 mg/L, 7.8 ± 1.0, and 0.02 ± 0.01 mg/L, respectively. Temperature was maintained at 24.4 ± 1.2°C. DO was measured twice daily using a portable DO meter (Model: EcoSense DO200A, Serial No: JC 07288, YSI Incorporated, OH 45387 USA). Ammonia and pH were monitored weekly using a test kit (patent no. ZL2010 2 0681385.X, Beijing Sunpu Biochem. Tech. Co., Ltd., Beijing, China).

### 2.3. Experimental Diets and Design

The experiment was conducted in a completely randomized design, containing four treatments and four replicates for each treatment. Four high-fiber (7.7% ± 0.3%), isonitrogenous (4.77% ± 0.01%), and isocaloric (3415.5 ± 31.1 kCal/kg) diets were formulated for the experiment. The diets were supplemented with graded levels of HY (Hilyses, ICC Animal Nutrition, Sao Paulo, Brazil) at 0.0 (control), 0.5 (HY0.5), 1.0 (HY1.0), and 2.0 (HY2.0) g/kg. All ingredients with large particles were pulverized and screened through a 60-mesh sieve. Then sieved ingredients, along with the vitamin–mineral premix and oil, were thoroughly mixed to form a uniform mixture. Pellets (2 mm) were extruded at 122 ± 5°C at a commercial feed manufacturing facility (Arman Feed Manufacturing Plant, Narsingdi, Bangladesh). The ingredients and proximate chemical compositions of the experimental diets are provided in Table 1.

### 2.4. Experimental Fish, Feeding Management, and Sampling Protocol

A total of 1500 healthy male Nile tilapia (*Oreochromis niloticus*) fingerlings, with an average body weight (ABW) of 5.0 g, were obtained from Meridian Hatchery, Mirsarai, Chattogram, Bangladesh. They were transported to the experimental site in oxygenated tanks and kept in the quarantine tank for a week. Following quarantine, the fish were distributed into four 1,100-L rearing tanks, raised, and acclimatized for 6 weeks, during which time, they were fed a commercial diet (Pushtiraj Feed, Narsingdi, Bangladesh).

After acclimation, 400 juveniles (10.0 ± 0.2 g) were evenly distributed into 16 tanks (25 fish per tank). The total biomass in each tank was equalized to achieve a coefficient of variation (CV) below 3%. From the following day, fish were hand-fed with experimental diets to apparent satiety three times daily (09:00 am, 12:00 pm, and 03:00 pm) over 14 weeks. After the end of each week, feed intake (FI) was measured by subtracting the amount of surplus feed from the supplied feed. Every 2 weeks, fish were sampled to assess growth performance. At the beginning of the trial, 32 fish (two fish/tank) were sampled and stored at −20°C in a freezer for initial body chemical composition analysis. At the end of the feeding trial, three fish from each tank (close to the ABW of that tank) were selected, ground to form a homogenized slurry, and stored at −20°C for final body chemical composition analysis.

### 2.5. Diet and Fish Sample Analysis

Both the diets and fish samples were analyzed for proximate compositions following the guidelines of the Association of Official Analytical Chemists (AOAC) [42]. In brief, moisture content was determined through oven drying at 105°C for 6 h (AOAC Method 934.01). The ash content was determined at 550°C for 4 h using a muffle furnace (Carbolite RHF 17/6S, Carbolite Ltd., England; AOAC Method 938.08). The crude protein content was evaluated by an auto Kjeldahl system (Model UDK159, VELP, Italy) according to AOAC Method 2001.11. The lipid content was assessed by a 16 h petroleum ether extraction using a Soxhlet Extractor (Model 6XL, Soxhlet Extractor Apparatus, Bakhshi Co., Tehran, Iran) following AOAC Method 2003.06. The crude fiber was estimated according to AOAC Method 978.10.

### 2.6. Growth Performance, Feed Utilization, and Biological Parameters

At the end of the trial, fish from each tank were weighed to determine ABW, body weight gain (BWG), FI, feed efficiency (FE), thermal-unit growth coefficient (TGC), and protein and energy efficiency ratios (PER and EER, respectively). Total feed utilization was calculated weekly throughout the experiment. Liver and viscera from five fish per tank were collected and weighed for hepatosomatic (HSI) and viscerosomatic (VSI) indices. The following equations were used to calculate the parameters:

  $$\begin{array}{c} \text{BWG }\left(g\right)=\text{Final BW }\left(\text{FBW},g\right)-\text{initial BW }\left(\text{IBW},g\right). \end{array}$$

  $$\begin{array}{c} \text{Feed intake }\left(g\right)=\text{Feed supplied }\left(g\right)-\text{feed surplus }\left(g\right). \end{array}$$

  $$\begin{array}{c} \text{FE}=\text{Biomass gain }\left(g\right)/\text{dry matter intake }\left(g\right). \end{array}$$

  $$\begin{array}{c} \text{Survival }\left(\%\right)=\left\{\left(\text{Final fish count}\right)|/|\left(\text{initial fish count}\right)\right\}\times 100\;. \end{array}$$

  $$\begin{array}{c} \text{TGC}=\left({\text{FBW}}^{\frac{1}{3}}-{\text{IBW}}^{\frac{1}{3}}\right)/\left(\sum \text{T}\times \text{days}\right). \end{array}$$

  $$\begin{array}{c} \text{PER }\left(\%\right)=\left\{\text{Weight gain }\left(g\right)\times 100\right\}/\text{protein intake }\left(g\right). \end{array}$$

  $$\begin{array}{c} \text{EER }\left(\%\right)=\left\{\text{Weight gain }\left(g\right)\times 100\right\}/\text{energy intake }\left(g\right). \end{array}$$

  $$\begin{array}{c} \text{HSI}=100\;\times \left\{\text{Liver wt}.\left(g\right)/\text{fish wt}.\left(g\right)\right\}. \end{array}$$

  $$\begin{array}{c} \text{VSI}=100\times \left\{\text{Viscera wt}.\left(g\right)/\text{fish wt}.\left(g\right)\right\}. \end{array}$$

### 2.7. Hematological Analysis

At the end of the feeding trial, two fish from each pen were anesthetized with clove oil containing 90% eugenol following the protocol described by Javahery et al. [43] for blood sample collection. The fish were immersed in clove oil solutions, typically prepared by diluting the oil in ethanol to facilitate emulsification. Upon exposure, the fish exhibited progressive loss of equilibrium and reduced opercular movement, indicating the onset of anesthesia. Hematological parameters including leukocyte count (WBC), erythrocyte count (RBC), hematocrit (HCT) level (%), hemoglobin (Hb) concentration (g/dL), mean corpuscular volume (MCV, fL), mean cell Hb (MCH, pg), mean corpuscular Hb concentration (MCHC, g/dL), and lymphocyte percentage (LYM%) were determined following standard protocols described by Zahan et al. [44]. In brief, blood samples were collected from the caudal peduncle of the fish by a 3-mL syringe. The needle was soaked in a 2% lithium heparin solution, commonly used as an anticoagulant in fish hematology, to prevent clotting during blood collection. After that, the blood was transferred into the ethylene diamine tetra acetic acid (EDTA) tube and stored at 4°C until analysis. The blood samples were then analyzed using a fully automated hematology analyzer (Model: DH36, Dymind Biotechnology, China).

### 2.8. Histology

Following the same protocol above, four fish from each treatment were anesthetized for collecting proximal intestinal tissue samples. After collecting, intestinal samples were gently rinsed with phosphate buffer saline to remove residual debris and stored in labeled glass bottles containing Bouin's fluid (a fixative composed of picric acid, formaldehyde, and acetic acid). Samples remained in the fixative for 24 h to ensure proper tissue preservation for histological analysis. The fixed samples were moved to 70% alcohol for preservation and kept at 4°C until histological examination. The fixed intestinal tissues were completely dehydrated by dipping into a graded series of alcohol. Then, molten wax was used to embed the samples. Trimming was performed on the prepared blocks, and the sections were cut at 10-μm thickness by a microtome machine. After staining with hematoxylin–eosin, the tissue was observed under a microscope (MCX100, Micros Austria, Sankt Veit an der Glan, Australia). Histological parameters of the intestine including villus length (VL), villus width (VW), and muscular thickness (MT) were measured using the image analysis application software Sigma Scan Pro5 (SPSS Inc., Chicago, IL, USA), as described by Bullerwell et al. [45]. The villus surface volume (SV) was calculated by using the following formula: SV = *π* × (VW/2)^2^ × VL. The histomorphological changes in the intestinal tissues were captured by a photomicroscope (AmScope 1000).

### 2.9. Gene Expression Analysis

Spleens from three randomly selected fish from each tank were collected for gene expression analysis. Total RNA was extracted from the spleens using Trizol/chloroform and a commercial RNA extraction kit (SERVA). Total RNA integrity (quality and quantity) for each sample was evaluated using 2% agarose gel electrophoresis and quantified using a NanoDrop 2000 Spectrophotometer (Nabi). The high-quality RNA samples were preserved at −80°C for subsequent analysis. Complementary DNA (cDNA) was synthesized from the total RNA samples (using 1 μg of RNA for each sample) by using the SensiFAST cDNA synthesis kit (Bioline) according to the manufacturer's protocol. The cDNA samples were then preserved at −20°C until further analysis.

Four different genes with different functional roles were selected for gene expression analysis including: genes related to growth and metabolism (insulin-like growth factor-1 [IGF-1] and glyceraldehyde-3-phosphate [G-3-P]), appetite (ghrelin), and immunity (hepcidin). Elongation factor 1 alpha was used as the reference gene as its suitability has been well-documented in numerous aquatic species [46–50]. Sequences of the target genes were collected from earlier studies on Nile tilapia [46]. Gene specific forward and reverse primer sequences are provided in Table 2 [51, 52]. Reactions were performed in 20 μL mixtures containing 10 μL of 2 × SensiFAST SYBR No-ROX Mix (Bioline), 3 μL of ultrapure water, 5 μL of template cDNA, and 1 μL of each forward or reverse primer. Reactions were then performed in triplicate for each sample using a real-time PCR system (Bio-Rad). At the end of each reaction, standard melt curve analysis was performed to confirm the amplification of a single-qPCR product. Finally, relative gene expression data were analyzed using the *Δ*Ct method [53–55] according to the following equation:

  
$$\begin{array}{c} \text{Relative gene expression }\left(R\right)=2^{-\left[\Delta \text{Ct target gene}-\Delta \text{Ct reference gene}\right]}. \end{array}$$

### 2.10. Statistical Analysis

All data were first examined for normality (Shapiro–Wilk) and homogeneity (Levene's test). One-way analysis of variance (ANOVA) was employed to identify significant differences among the treatments, followed by Duncan's multiple range test (DMRT) to compare the mean values between treatments using SPSS 22.0 (IBM, USA). A linear regression analysis was performed to evaluate the dose–response effects of HY. A third-order polynomial regression analysis was applied to determine the optimal inclusion level of HY, based on dose–response trends for performance indices such as FE, PER, EER, and feed cost per unit gain. The four graded HY levels provided adequate resolution for fitting a nonlinear model, consistent with standard practice in aquaculture nutrition trials. The model yielded high coefficients of determination (*R*^2^ = 1.0), and the local maxima of the fitted curves were used to identify optimum supplementation ranges. Finally, a principal component analysis (PCA) based on Pearson correlation was also performed to explore the parameters significantly related to the dietary HY level.

## 3. Results

### 3.1. Growth Performance, Feed Utilization, and Body Composition

Table 3 presents the effects of dietary supplementation of HY on the growth performance and feed utilization during the feeding trial. There were no significant differences (*p* > 0.05) among the dietary treatments in most parameters, except for the EER and HSI (*p* < 0.05). Notably, the HSI was much lower in fish-fed HY1.0 diets compared to those fed the control or HY2.0 diets. Despite no significant differences among the treatments, there was an increasing trend in ABW, BWG, TGC, EER, and PER with the increasing level of dietary HY. Linear regression further confirmed a significantly positive trend for FE, EER, and PER with the HY level. Interestingly, feed cost per kg of fish produced declined linearly with HY supplementation.

Table 4 displays the proximate body composition of tilapia-fed experimental diets. Despite no differences (*p* > 0.05) among the treatment groups, fish fed the HY2.0 diet exhibited higher dry matter and crude protein content than the other treatments. Notably, both parameters showed a significantly linear trend in response to the increasing dietary HY level.

### 3.2. Nutrient Deposition, Energy Retention, and Their Efficiency

Table 5 shows the assimilated protein and lipid deposition, retained energy, and their respective retention efficiencies. Both protein deposition (PD) and protein retention efficiency (PRE) were considerably higher in fish fed the HY2.0 diets than in the other treatment groups. Importantly, these parameters, along with energy retention efficiency (ERE), improved linearly (*p* < 0.05) with increasing dietary levels of HY.

### 3.3. Hematological Parameters

The hematological responses of *O. niloticus* to dietary HY supplementation are summarized in Table 6. Among the measured parameters, HCT was the only parameter significantly affected by the treatments, with the HY0.5 group exhibiting the highest value, while the HY1.0 and HY2.0 groups showed the lowest. Linear regression with the dietary levels of HY also showed a similar pattern, where only HCT showed a significant linear relationship with HY level. In contrast, other hematological indices including RBC, WBC, Hb, MCV, MCH, and MCHC remained statistically unaffected by the dietary treatments.

### 3.4. Gut Histology

**Figure 1:**
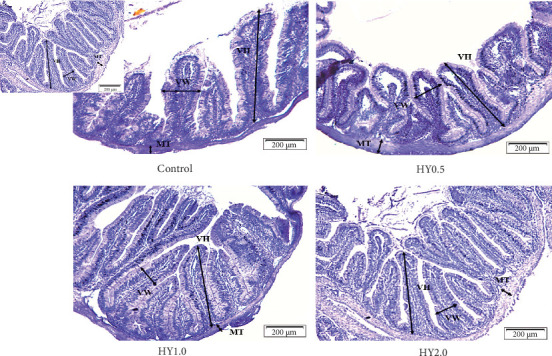
Cross section of the proximal intestines of fish fed the treatment diets. HY0.5, hydrolyzed yeast 500 mg/kg; HY1.0, hydrolyzed yeast 1 g/kg; HY2.0, hydrolyzed yeast 2 g/kg.

Table 7 shows the gut histological parameters of fish fed the treatment diets. VL (µm) and villus SV (mm^3^) were the highest in fish fed the HY2.0 diets and significantly higher than those fed the control diet. Both parameters also showed a significant linear trend with dietary HY levels. There were no notable variations (*p* > 0.05) observed in terms of VW (µm) and MT (µm) among the treatment groups, despite a higher value for fish fed HY2.0 diets than those fed the other diets. The corresponding histology figures are presented in Figure 1.

### 3.5. Gene Expression

**Figure 2:**
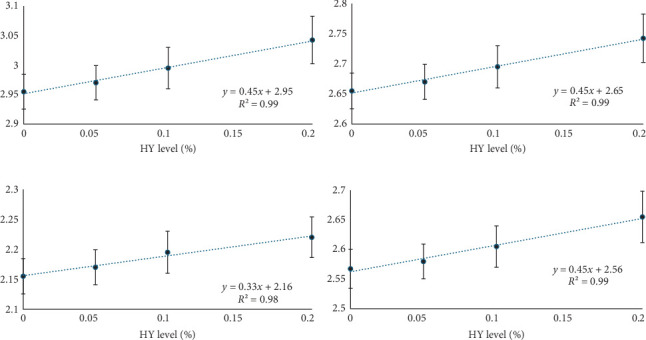
Regression of HY level (%) with target genes expression values: (A) IGF-I (insulin-like growth factor-1), (B) G-3-P (glyceraldehyde-3-phosphate), (C) ghrelin, and (D) hepcidin.

The expression of all target genes (IGF-1, G-3-P, ghrelin, and hepcidin) was upregulated in fish receiving HY-containing diets compared to those fed the control diet (Table 8). Furthermore, a significant linear trend in the expression of all four candidate genes was observed with the increasing level of dietary HY (Figure 2).

### 3.6. PCA

**Figure 3:**
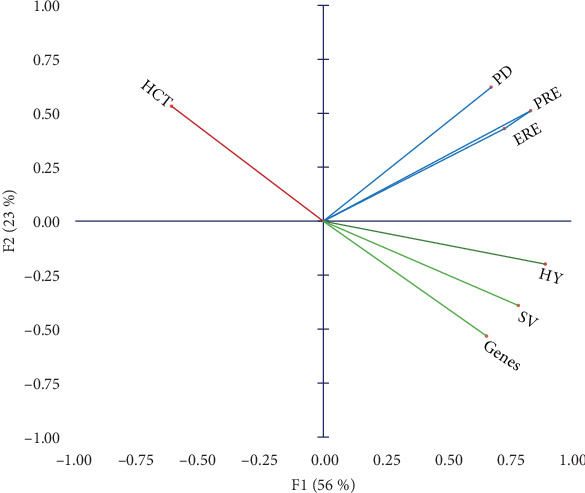
Principal component analysis (PCA) of selected variables. ERE, energy retention efficiency; HCT, hematocrit; HY, hydrolyzed yeast; PD, protein deposition; PRE, protein retention efficiency; SV, surface volume. Genes, average values of the selected genes. The first two components combined explain 78.85% of the variations.

Parameters selected for PCA were HY level, PD, PRE, ERE, HCT, villus SV, and combined gene expression parameter (Genes). All parameters were significantly correlated with HY where HCT was negatively correlated (*r* = −0.606; Figure 3).

### 3.7. The Optimal Dose

**Figure 4:**
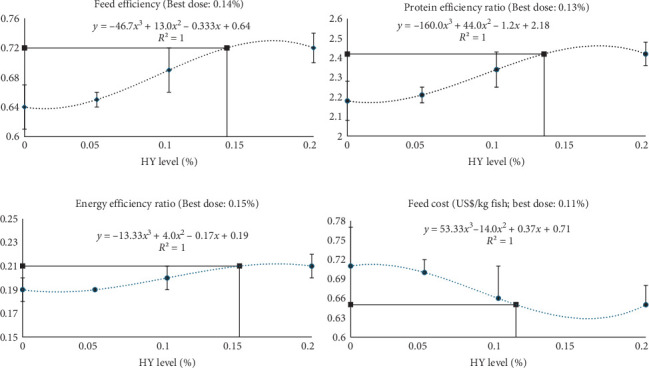
Regression of HY level (%): (A) feed efficiency, (B) protein efficiency ratio, (C) energy efficiency ratio, and (D) feed cost. The arrow indicates the predicted optimal inclusion level (asymptote) based on the polynomial regression model.

The third-order polynomial regression analysis of FE, PER, EER, and feed cost against the HY level showed that the optimum inclusion level of HY was 0.112%–0.151% (i.e., 1.12–1.51 g), respectively (Figure 4).

## 4. Discussion

Quality feed ingredients are costly, and the market is volatile, while cost-effective ingredients may contain anti-nutritional factors, be usually poorly digestible, and thus, may have negative impacts on animal health and the surrounding environment. The uncertainty in managing these ever-changing situations, including raw material quality, supply inconsistencies, high market price, and environmental challenges, has, therefore, led to an increased use of functional feed additives, which includes enzymes, prebiotics, probiotics, and gut acidifiers. While live yeasts are probiotic, inactivated yeasts function as prebiotics with immune-modulatory actions due to their high MOS and β-glucan contents, two major components of their cell wall. As yeasts can be easily genetically manufactured, they are also used as industrial chassis to produce important secondary metabolites, from vitamins to minerals to fatty acids to enzymes on demand [56].

The HY used in this study is an inactivated, HY derived from the fermentation of sugarcane for bioethanol production using *Saccharomyces cerevisiae*. The results revealed no significant differences among the treatments in most growth performance parameters except the EER and HSI. However, a significant positive linear trend was evident with the increasing dosages of the dietary HY in FE (*t* = 2.535), PER (*t* = 2.572), and EER (*t* = 3.349). Regression analysis indicated that the optimum inclusion level of HY in the Nile tilapia diet is 1.12 to 1.51 g per kg of feed to induce maximum growth with better nutrient efficiency along with lower feed cost. These findings are consistent with those reported by El-Naby et al. [20] and Sheikhzadeh et al. [57], who observed enhanced FE following dietary inclusion of the same HY product. Additionally, El-Naby et al. [20] documented progressive improvements in PER and EER with increasing dietary HY levels, identifying 2.0 g/kg as the optimal supplementation dose. Mainly due to this improvement in the FE, the feed cost to produce 1 kg of fish also reduced linearly from 0.71US$ for those who fed the control diet to 0.66 and 0.65 US$ for those fed HY1.0 and HY2.0 diets, respectively (*t* = −2.074; *p* = 0.057). HY contains a proprietary blend of bioactive compound including β-glucans, free nucleotides, glutamine, peptides, and MOS, formulated from the enzymatic fermentation of *Saccharomyces cerevisiae*. These components, known for their functional properties, may contribute to enhanced growth performance by acting as prebiotics and immunostimulants [20]. Whether they function individually or synergistically, they can support digestive processes, stimulate enzyme activity, improve gut microbiota, and optimize mucosal health, collectively promoting better nutrient absorption, feed utilization, and overall growth in aquatic species [58–60]. Thus, the use of HY in this study not only demonstrated functional benefits in nutrient efficiency and feed cost reduction but also reflects a broader sustainability strategy. As a byproduct of sugarcane fermentation for bioethanol production, HY exemplifies circular economy principles by transforming agro-industrial waste into a high-value aquafeed ingredient. This valorization reduces environmental burden and supports resource-efficient feed formulation, aligning with recent efforts to integrate circularity into aquaculture nutrition frameworks [39–41].

PD (*t* = 2.179) and PRE (*t* = 3.056) in this study also increased linearly (*p* < 0.05) with the increasing HY dosages. Similar findings were reported in other studies involving both activated [61] and inactivated yeast [62]. El-Naby et al. [20] also reported an elevated PD in the tissue composition of Nile tilapia due to dietary HY supplementation. Among the blood parameters, HCT exhibited significant differences among treatments, showing a decreasing trend with higher levels of HY. In the gut histology, VL and nutrient absorption capacity, depicted as villus SV, both increased significantly with HY supplementation. Correspondingly, El-Naby et al. [20] observed notable enhancements of intestinal morphology, including elevated VL and VW, a higher count of mucosal fold and increased muscular layer thickness. These findings are in contrast with the ones reported by Sheikhzadeh et al. [57], who reported no significant changes in those parameters using the same HY. Perhaps, a wider range of HY dosage (0–2.0 g/kg) used in this study than those (0–0.5 g/kg) used by Sheikhzadeh et al. [57] is the reason for finding the significant trend in these parameters.

The candidate genes in this study were selected due to their inferred functional roles in several earlier studies [46, 63–65]. They are genes related to growth and metabolism (IGF-1 and G-3-P), hunger regulation (ghrelin), and immunity or defense against pathogens (hepcidin). In animals, information about metabolic status arrives in the brain in the form of a complex milieu of circulating signaling factors, including glucose, fatty acids, ghrelin, leptin, and insulin. Ghrelin is a stomach-derived hormone usually known for appetite regulation that regulates several other metabolic functions including the release of growth hormone (GH) [66], adiposity [67], gastric motility, and gut–microbiome modulation [68]. The regulation of appetite may be influenced by H_2_S, produced endogenously by the gut microbiome, which directly regulates ghrelin secretion, which in turn affects the appetite of mice [68]. While appetite is regulated by ghrelin, leptin, and insulin, somatic growth is mainly regulated by GH, insulin, IGFs I and II, and thyroid hormones [69, 70]. IGF mRNA is found in all life stages in fish, from unfertilized eggs to adults. IGFs in fish stimulate myogenic cell proliferation, differentiation, and protein synthesis through the MAPK/ERK and PI3K/AKT/TOR signaling pathways, also reducing protein degradation and atrophy via the PI3K/AKT/FOXO signaling pathway [71]. Overexpression of IGF-1 in skeletal muscle promotes myofiber hyperplasia and cellularity changes, which elicit alterations in the body's energy metabolism and skeletal muscle growth [72]. In this study, a linear increase in the expression of the growth-related genes with increasing levels of HY was observed. The finding is in accordance with the findings of similar small peptide-rich products such as fish hydrolysates [73]. Midhun et al. [74] reported modulation of digestive enzymes and GHs when supplemented with graded levels of curcumin. Like curcumin, small peptides, present in both fish hydrolysates and yeast, have been shown to increase the production of digestive enzymes. Consistent with this, Heidarieh et al. [75] reported significant increases in trypsin and amylase activities in juvenile fish following HY supplementation. This raises an interesting question: could there be a relation between the secretion of digestive enzymes such as α-amylase, protease, and lipase to the expression of IGF-1 or other GHs? However, Odu-Onukosi et al. [76] did not find any significant changes in the expression of G-3-P gene expression with the addition of the graded level of autolyzed yeast despite significant improvement in the growth. Several factors such as life stages (i.e., juvenile in this study vs. early life stage), yeast processing method (enzymatically hydrolyzed in this study vs. mechanically autolyzed), or the tissue sampled (i.e., spleen in this study vs. mid-intestine) could play a role in the differences in the G-3-P gene expression between the studies. Regulation of gene expressions within the intestinal epithelium is complex and controlled by various signaling pathways that could be significantly different than the ones in the spleen [77]. Although mostly known for iron homeostasis, studies on hepcidin in teleost fish have confirmed the relevance of the molecule in the defense system against pathogenic microorganisms [78, 79]. Hepcidin-20 induces the expression of pro-inflammatory cytokines TNF-α and IL1-β in the head-kidney of European sea bass, but not the anti-inflammatory cytokine IL-10 expression [80]. However, not all hepcidins possess anti-microbial properties. For example, hepcidin-25 in European sea bass [80] and TH1-5 and TH2-2 in tilapia [81] did not show any antimicrobial activity. Hsieh et al. [81] also reported that only TH2-3 demonstrated antimicrobial activity in tilapia when challenged with *Vibrio vulnificus* and *Streptococcus agalactiae*, with similar effects reported in grouper by Ting et al. [82]. The upregulation of hepcidin and IGF-1 in response to HY supplementation suggests a dual role of HY in enhancing both immune response and growth performance in Nile tilapia, potentially aiding in inflammation control, pathogen defense, and muscle development. Given that the fish were raised in a suboptimal temperature environment, which can induce physiological stress, this genetic modulation suggests that HY may have contributed to a more favorable physiological environment, promoting not only growth but also immune and intestinal health. This could be an underlying mechanism for the improvement in the observed zootechnical performance.

## 5. Conclusion

In conclusion, this study highlights the potential of using HY derived from sugarcane fermentation for bioethanol production as a functional feed additive in aquaculture. Despite limited differences in growth performance parameters, a notable linear improvement was observed in feed conversion efficiency, protein and energy utilization, and cost-effectiveness with increasing dosages of HY. Additionally, enhanced PD, retention efficiency, and gut histology parameters, coupled with significant trends in the expression of growth-related genes, suggest the multifaceted benefits of HY in promoting nutrient utilization, growth, and metabolic regulation. These findings, combined with its immune-modulatory effects, reinforce the promise of HY as a sustainable feed additive to address the challenges of fluctuating raw material quality and environmental pressures in aquaculture. Future research could explore the underlying mechanisms in greater detail to optimize their application across different species and production systems.

## Figures and Tables

**Table 1:** Ingredients and proximate composition (%) of the experimental diets.

Ingredients (%)	Control	HY0.5	HY1.0	HY2.0
Maize	4.05	3.00	4.05	3.65
Rice polish	15.00	14.90	14.55	14.55
De-oiled rice bran	23.70	25.20	24.30	25.10
Wheat flour	5.45	5.30	5.15	5.15
Rapeseed meal, 37% CP	27.30	27.35	27.30	27.10
Soybean meal, 49% CP	19.00	18.65	18.90	18.80
Limestone	1.50	1.50	1.55	1.35
Monocalcium phosphate	0.10	0.10	0.10	0.10
Salt	0.45	0.45	0.45	0.45
Choline chloride	0.03	0.03	0.03	0.03
L-lysine	0.70	0.70	0.70	0.70
DL-methionine	0.32	0.32	0.32	0.32
Fish premix^a^	0.10	0.10	0.10	0.10
Rice bran oil	2.30	2.35	2.40	2.40
Hilyses	0.00	0.05	0.10	0.20
Total	100.00	100.00	100.00	100.00
Formulation cost (US$/kg)	$0.42	$0.42	$0.42	$0.43
Proximate chemical composition (dry matter basis)				
Dry matter (%)	91.8	90.8	91.9	90.4
Crude protein (%)	29.7	29.9	29.8	29.9
Crude fat (%)	6.3	6.7	7.5	7.6
Crude ash (%)	10.1	10.0	10.2	10.1
Total carbohydrate	45.7	45.2	44.4	42.8
Gross energy (kCal/g; calculated)	3.73	3.77	3.85	3.86
Crude fiber (%)	7.4	7.6	8.0	8.0

*Note:* HY0.5, hydrolyzed yeast 500 mg/kg; HY1.0, hydrolyzed yeast 1 g/kg; HY2.0, hydrolyzed yeast 2 g/kg.

^a^Each kilogram of premix contains the following: vitamin A, 0.8 g; vitamin D3, 0.08 g; vitamin E, 20 g; vitamin K3, 18.6 g; vitamin B1, thiamine hydrochloride, 10 g; riboflavin B2, 12.5 g; vitamin B6, 8 g; vitamin C, 400 mg; calcium pantothenate B5, 20 g; folic acid, 3.2 g; biotin, 3 g; inositol, 406 g; niacin, 12 g; calcium lactate, 500 g; sodium dihydrogen phosphate, 100 g; ferrous sulfate, 20 g; sodium chloride, 20 g; anhydrous magnesium sulfate, 100 g; aluminum chloride, 0.6 g; zinc sulfate, 20 g; manganese sulfate, 4 g; copper sulfate, 2 g; potassium chloride, 40 g; cobalt chloride, 2 g; potassium iodate, 0.6 g; sodium selenite, 0.004 g.

**Table 2:** Gene-specific forward (F) and reverse (R) primer sequences with specific details [51, 52].

Candidate gene	Primer sequence (5^ʹ^–3^ʹ^)	References
Insulin-like growth factor I (IGF-I)	F: TTGTCTGTGGAGAGCGAGGCT	[51]
	R: CAGCTTTGGAAGCAGCACTCGT	
Glycerol-3-phosphate (G-3-P)	F: CTATCCATGGAGCCTCAGGT	[51]
	R: CTTCTTGAGCGTGGCAATAA	
Hepcidin	F: GTCTGTCAAGGATAAGCGCTG	[52]
	R: ACTCTGGAGCTGGATGTTGA	
Gherlin	F: TAATGGGAGAGGGAAGATGG	[52]
	R: CTCTGCGATGTAATTCAGGA	

**Table 3:** Growth performance, feed utilization, and somatic indices of *Oreochromis niloticus* fed the experimental diets.

Parameters	ANOVA					Regression vs. HY (%)	
	Control	HY0.5	HY1.0	HY2.0	*p*-Value	*t*	*p*-Value
FBW (g/fish)	42.2 ± 1.97	44.7 ± 1.60	43.5 ± 0.95	45.5 ± 1.04	0.427	1.430	0.175
WG (g/fish)	32.3 ± 1.93	34.7 ± 1.61	33.5 ± 1.03	35.5 ± 1.04	0.461	1.385	0.188
FI (g/fish)	49.7 ± 1.48	52.7 ± 3.19	48.1 ± 1.05	49.1 ± 0.92	0.387	−0.725	0.481
FE	0.65 ± 0.05	0.65 ± 0.02	0.70 ± 0.05	0.72 ± 0.03	0.148	2.535	0.024
TGC	0.65 ± 0.04	0.68 ± 0.04	0.67 ± 0.03	0.69 ± 0.02	0.437	1.475	0.162
PER	2.18 ± 0.17	2.21 ± 0.07	2.34 ± 0.15	2.42 ± 0.11	0.154	2.572	0.022
EER	0.19^b^ ± 0.01	0.19^b^ ± 0.01	0.20^a,b^ ± 0.01	0.22^a^ ± 0.01	0.050	3.349	0.005
HSI (%)	2.89^a^ ± 0.13	2.33^a,b^ ± 0.30	1.51^b^ ± 0.70	2.56^a^ ± 0.29	0.011	−0.643	0.531
VSI (%)	10.6^a,b^ ± 1.1	11.8^a^ ± 0.4	10.0^b^ ± 1.1	11.4^a,b^ ± 0.6	0.106	0.510	0.618
Survival (%)	98.0^a,b^ ± 2.0	97.0^a^ ± 1.7	100.0^b^ ± 0.0	96.0^a,b^ ± 4.0	0.262	−0.183	0.858
Feed cost (US$/kg fish)	0.71 ± 0.06	0.70 ± 0.02	0.67 ± 0.05	0.65 ± 0.03	0.282	−2.074	0.057

*Note:* HY0.5, hydrolyzed yeast 500 mg/kg; HY1.0, hydrolyzed yeast 1 g/kg; HY2.0, hydrolyzed yeast 2 g/kg. The different letters in superscript in a row denote significant differences at *p* < 0.05.

Abbreviations: EER, energy efficiency ratio; FBW, final body weight; FE, feed efficiency; FI, feed intake; HSI, hepatosomatic index; PER, protein efficiency ratio; TGC, thermal-unit growth coefficient; VSI, viscerosomatic index; WG, weight gain.

**Table 4:** Final body composition of fish fed the experimental diets.

Parameters	ANOVA					Regression vs. HY (%)	
	Control	HY0.5	HY1.0	HY2.0	*p*-Value	*t*	*p*-Value
DM (%)	35.3 ± 0.37	35.2 ± 0.32	36.4 ± 1.54	37.0 ± 1.92	0.269	2.045	0.060
CP (%)	16.0 ± 0.29	16.4 ± 0.48	15.6 ± 1.05	17.4 ± 0.91	0.068	1.907	0.077
CL (%)	11.35 ± 0.09	10.71 ± 0.39	11.77 ± 0.89	10.75 ± 0.70	0.132	0.655	0.523
Ash (%)	9.62 ± 1.00	9.26 ± 0.90	8.87 ± 1.14	8.94 ± 1.67	0.868	−0.748	0.467
GE (kCal)	56.16 ± 5.45	58.86 ± 4.31	59.09 ± 5.51	62.31 ± 10.81	0.623	1.412	0.180

*Note:* HY0.5, hydrolyzed yeast 500 mg/kg; HY1.0, hydrolyzed yeast 1 g/kg; HY2.0, hydrolyzed yeast 2 g/kg.

Abbreviations: CL, crude lipid; CP, crude protein; DM, dry matter; GE, gross energy.

**Table 5:** Protein and lipid deposition, retained energy, and their retention efficiency in fish fed the experimental diets.

Parameters	ANOVA					Regression vs. HY (%)	
	Control	HY0.5	HY1.0	HY2.0	*p*-Value	*t*	*p*-Value
PD (g)	4.72 ± 0.42	5.26 ± 0.49	4.74 ± 0.58	5.87 ± 0.72	0.087	2.179	0.047
LD (g)	4.31 ± 0.38	4.30 ± 0.29	4.64 ± 0.49	4.43 ± 0.50	0.731	0.498	0.627
RE (kCal)	94.1 ± 15.7	104.1 ± 13.7	99.1 ± 7.3	106.3 ± 9.2	0.613	1.084	0.297
PRE (%)	29.3 ± 2.0	30.4 ± 1.3	30.3 ± 3.2	36.2 ± 4.3	0.052	3.056	0.009
LRE (%)	126.3 ± 8.1	104.1 ± 13.7	99.1 ± 7.3	106.3 ± 9.2	0.139	−1.963	0.070
ERE (%)	32.3 ± 2.1	31.6 ± 1.4	35.0 ± 3.0	36.2 ± 4.0	0.200	2.154	0.049

*Note:* HY0.5, hydrolyzed yeast 500 mg/kg; HY1.0, hydrolyzed yeast 1 g/kg; HY2.0, hydrolyzed yeast 2 g/kg.

Abbreviations: ERE, energy retention efficiency; LD, lipid deposition; LRE, lipid retention efficiency; PD, protein deposition; PRE, protein retention efficiency; RE, retained energy.

**Table 6:** Hematological parameters of fish fed the experimental diets.

Parameters	ANOVA					Regression vs. HY (%)	
	Control	HY0.5	HY1.0	HY2.0	*p*-Value	*t*	*p*-Value
RBC (×10^6^/µL)	1.83 ± 0.21	1.85 ± 0.08	1.80 ± 0.12	1.76 ± 0.15	0.889	−0.737	0.474
WBC (×10^3^/µL)	49.9 ± 5.3	54.03 ± 2.55	51.87 ± 5.19	58.08 ± 9.90	0.460	1.540	0.146
Hb (g/dL)	7.01 ± 0.95	7.59 ± 0.85	6.54 ± 0.35	6.30 ± 0.94	0.273	−1.551	0.143
HCT (%)	26.06^a,b^ ± 4.45	27.95^a^ ± 3.10	20.93^b,c^ ± 2.56	19.90^c^ ± 1.89	0.023	−2.851	0.013
MCV (fL)	143.6^a,b^ ± 7.4	150.8^a^ ± 12.6	116.0^b^ ± 13.8	132.0^a,b^ ± 29.9	0.295	−0.969	0.349
MCH (pg)	38.30 ± 3.98	41.06 ± 3.59	36.38 ± 0.77	35.74 ± 1.89	0.247	−1.447	0.170
MCHC (g/dL)	27.45^a,b^ ± 2.48	27.30^b^ ± 0.56	32.08^a^ ± 3.51	27.81^a,b^ ± 3.33	0.153	0.393	0.700
LYM (%)	91.74^a,b^ ± 0.87	92.48^a^ ± 0.60	92.05^ab^ ± 0.56	91.10^b^ ± 0.89	0.197	−1.443	0.171

*Note:* HY0.5, hydrolyzed yeast 500 mg/kg; HY1.0, hydrolyzed yeast 1 g/kg; HY2.0, hydrolyzed yeast 2 g/kg. The different letters in superscript in a row denote significant differences at *p* < 0.05.

Abbreviations: HCT, hematocrit; Hgb, hemoglobin; LYM, lymphocytes; MCH, mean corpuscular hemoglobin; MCHC, mean corpuscular hemoglobin concentration; MCV, mean corpuscular volume; RBC, red blood cell; WBC, white blood cell.

**Table 7:** Intestinal histological parameters for fish fed the experimental diets.

Parameters	ANOVA					Regression vs. HY (%)	
	Control	HY0.5	HY1.0	HY2.0	*p*-Value	*t*	*p*-Value
VL (µm)	504^b^ ± 80.71	621^a,b^ ± 155.02	679^a^ ± 85.16	792^a^ ± 89.60	0.091	4.113	0.001
VW (µm)	226 ± 19.08	225.5 ± 11.34	221.5 ± 10.49	244.8 ± 29.04	0.349	1.242	0.235
MT (µm)	69.2^b^ ± 20.11	92.7^a,b^ ± 30.54	80.3^a,b^ ± 18.31	97.6^a^ ± 15.65	0.301	1.493	0.158
SV (mm^3^)	0.021^b^ ± 0.006	0.025^b^ ± 0.005	0.026^b^ ± 0.002	0.038^a^ ± 0.010	0.018	3.560	0.003

*Note:* HY0.5, hydrolyzed yeast 500 mg/kg; HY1.0, hydrolyzed yeast 1 g/kg; HY2.0, hydrolyzed yeast 2 g/kg. The different letters in superscript in a row denote significant differences at *p* < 0.05.

Abbreviations: MT, muscle thickness; SV, surface volume; VL, villi length; VW, villi width.

**Table 8:** Expression of target genes in fish fed the experimental diets.

Parameters	ANOVA					Regression vs. HY (%)	
	Control	HY0.5	HY1.0	HY2.0	*p*-Value	*t*	*p*-Value
IGF-1	2.96^b^ ± 0.03	2.97^b^ ± 0.03	3.00^a,b^ ± 0.04	3.04^a^ ± 0.04	0.038	3.648	0.003
G-3-P	2.66^b^ ± 0.03	2.67^b^ ± 0.03	2.70^a,b^ ± 0.04	2.74^a^ ± 0.04	0.038	3.648	0.003
Ghrelin	2.16^b^ ± 0.03	2.17^a,b^ ± 0.03	2.20^a,b^ ± 0.04	2.22^a^ ± 0.03	0.120	2.848	0.013
Hepcidin	2.57^b^ ± 0.03	2.58^b^ ± 0.03	2.61^a,b^ ± 0.04	2.66^a^ ± 0.04	0.047	3.494	0.004

*Note:* HY0.5, hydrolyzed yeast 500 mg/kg; HY1.0, hydrolyzed yeast 1 g/kg; HY2.0, hydrolyzed yeast 2 g/kg. The different letters in superscript in a row denote significant differences at *p* < 0.05.

Abbreviations: IGF-1, insulin-like growth factor-1; G-3-P, glyceraldehyde-3-phosphate.

## Data Availability

The data that support the findings of this study are available upon request from the corresponding author. The data are not publicly available due to privacy or ethical restrictions.
